# In vitro and in vivo validation of a new three‐dimensional fringe projection‐based device (AEVA‐HE) dedicated to skin surface mapping

**DOI:** 10.1111/srt.13209

**Published:** 2023-02-07

**Authors:** Ayet Shaiek, Muriel Monot, Virginie Rubert, Celine Cornillon, Marco Vicic, Frederic Flament, Geraldine Decocq, Jean‐Jacques Servant, Gabriel Koeller, Christelle Lille

**Affiliations:** ^1^ L'Oréal Research and Innovation Paris France; ^2^ Eotech Marcoussis France

**Keywords:** AEVA‐HE, DermaTOP, fringe projection, validation, vitro, vivo

## Abstract

**Background:**

Aging signs are much visible on the surface of the skin that presents different changes: cheeks start to sag, more and deeper wrinkles appear, and pigmentation spots increase. Face diagnostic to recommend products includes assessing cutaneous micro‐relief or the micro‐depressive network on the face. Furthermore, there is an increasing demand for clinical and instrumental methods to prove the efficacy of anti‐aging treatments. As a result, very accurate and sensitive three‐dimensional (3D) devices are developed and validated to measure and quantify aging skin and to catch fine anti‐aging products acting on wrinkles and fine lines.

**Methods:**

AEVA‐HE, a non‐invasive 3D method based on fringe projection technology, is used to robustly characterize the skin micro‐relief from a full‐face acquisition and from multiple extracted zones of interest. In vitro and in vivo studies are conducted to assess the reproducibility of this system and its precision toward a standard fringe projection system, DermaTOP.

**Results:**

The AEVA‐HE successfully measured micro‐relief and wrinkles and demonstrated the reproducibility of measurements.  AEVA‐HE parameters were found highly correlated to DermaTOP.

**Conclusion:**

The present work illustrates the performance of the AEVA‐HE device and its dedicated software kit as a precious tool for quantifying the major characteristics of wrinkles appearing with age and thus offers a high potential for assessing the effect of anti‐wrinkling products.

## 1 INTRODUCTION

Aging skin is mostly characterized by a progressive alteration of the structural components of the dermal connective tissue. These deep modifications consequently lead to the occurrence of external skin signs such as wrinkles, loss of elasticity, a rougher texture, and heterogeneous pigmentation.^1^ Hence, over a lifetime, the skin will present changes in both appearance and structure.

Apart from unavoidable chronological and intrinsic processes, several external factors such as gravity, sun/ultraviolet exposure, pollution, including lifestyle factors (diet, tobacco, illness, or stress).^2^ In short, all these possibly mixed factors contribute to exacerbating the skin aging process.

From a cosmetic viewpoint, improving skin firmness, preventing or delaying the occurrence of wrinkles, and decreasing their severities are key objectives to which moisturizing, protecting, and nourishing the skin are important pillars.^4^

With aging, the skin texture is mainly rated, at a glance, by wrinkles that progressively arise from atrophy of the various skin layers, dermal elastosis, and facial expressions. Micro‐lines or permanent folds, related to facial expressions, such as the crow's feet, the contour of the lips, and the forehead, will therefore deepen over years, from fine lines to wrinkles. This skin micro‐relief, hardly visible to the naked eye, can only be studied through highly resolving technologies such as two‐dimensional (2D) Imagery systems^6^, ^7^ and 3D high‐resolution imagery devices. Replica methods associated with an imagery acquisition and analysis system are commonly used to study the formation of wrinkles during aging and their regression induced by an anti‐wrinkle product.^8^, ^9^ Many hand‐held 3D systems of high resolution are proposed to the market and are able to measure numerous skin features such as pores, wrinkles, and depressions/elevations of the skin obtained from single or multiple acquisitions. These instruments could capture the skin typography on contact with the skin like the Antera 3D.^10^, ^11^ ANTERA 3D (Miravex) relies on multidirectional illumination obtained by LEDs of different wavelengths which makes it advanced in the qualitative evaluation of various dermatologic conditions.^12^, ^13^ Non‐contact devices comprise the Artec systems.^14^, ^15^ These hand‐held systems, although suitable for capturing small objects (small field of view [FOV]) of complex geometry, showed nevertheless a higher variability when compared to fringe projection systems ^16^. The principle of fringe projection devices comprises a projector of several phase‐shifted stripe patterns deformed by the skin topography and a camera unit, positioned at a different angle from the projector that captures the shifted fringe images. The generated images are used to create a 3D mapping of the skin topography, and then collect several features such as profile roughness/deepness, wrinkle features, and volume parameters. Fringe Projection has been adapted and used in many clinical studies as a non‐contact device to assess the efficacy of anti‐aging cosmetic preparations targeting the volume of wrinkles. The most commonly used system relying on this technology is the DermaTOP (Eotech).^17^, ^18^

In fact, these high‐resolution 3D devices and their dedicated software allow a robust and precise characterization of the skin relief^19^, ^20^ and, for example, are thus able to demonstrate the effect of a smoothing product effect on a less marked relief or evaluate the degree of efficacy of eliminating crow's feet by means of injection of botulinum toxin.^21^

When dealing with the assessment of product effects on wrinkles, the major challenge is the ability of the system to reproduce the same measurement of the subject at different time points, due to reliable repositioning and the ability of the algorithms to extract the exact zones at these different time points. The pertinence of physical parameters generated by the 3D analysis is directly impacted by both the 3D device performance and by environmental factors (like repositioning) and skin conditions such as shine.

The recently developed AEVA‐HE system,^22^ based on a patented fringe projection unit combined with stereo imaging techniques, significantly improves its robustness and reproducibility capability to capture smartly, in one acquisition, the full‐face details. Furthermore, a 3D data analysis could be holistic, by giving a global evaluation of aging signs or multi‐zones (crow's foot, nasolabial fold, frown lines, under‐eye bags, ptosis, etc.), thus allowing a multi‐parametric evaluation of the face. The method currently in use focuses on surface roughness (wrinkles, pores) and volume measurement of the sagging feature. In this paper, we present evaluation studies of the performance of the new AEVA‐HE system in terms of repeatability and its precision in characterizing skin relief. One ground truth system is considered: DermaTOP. The results of this validation phase are the objects of the present paper.

## 2 MATERIALS AND METHODS

### 2.1 AEVA‐HE and DermaTOP technologies

**FIGURE 1:**
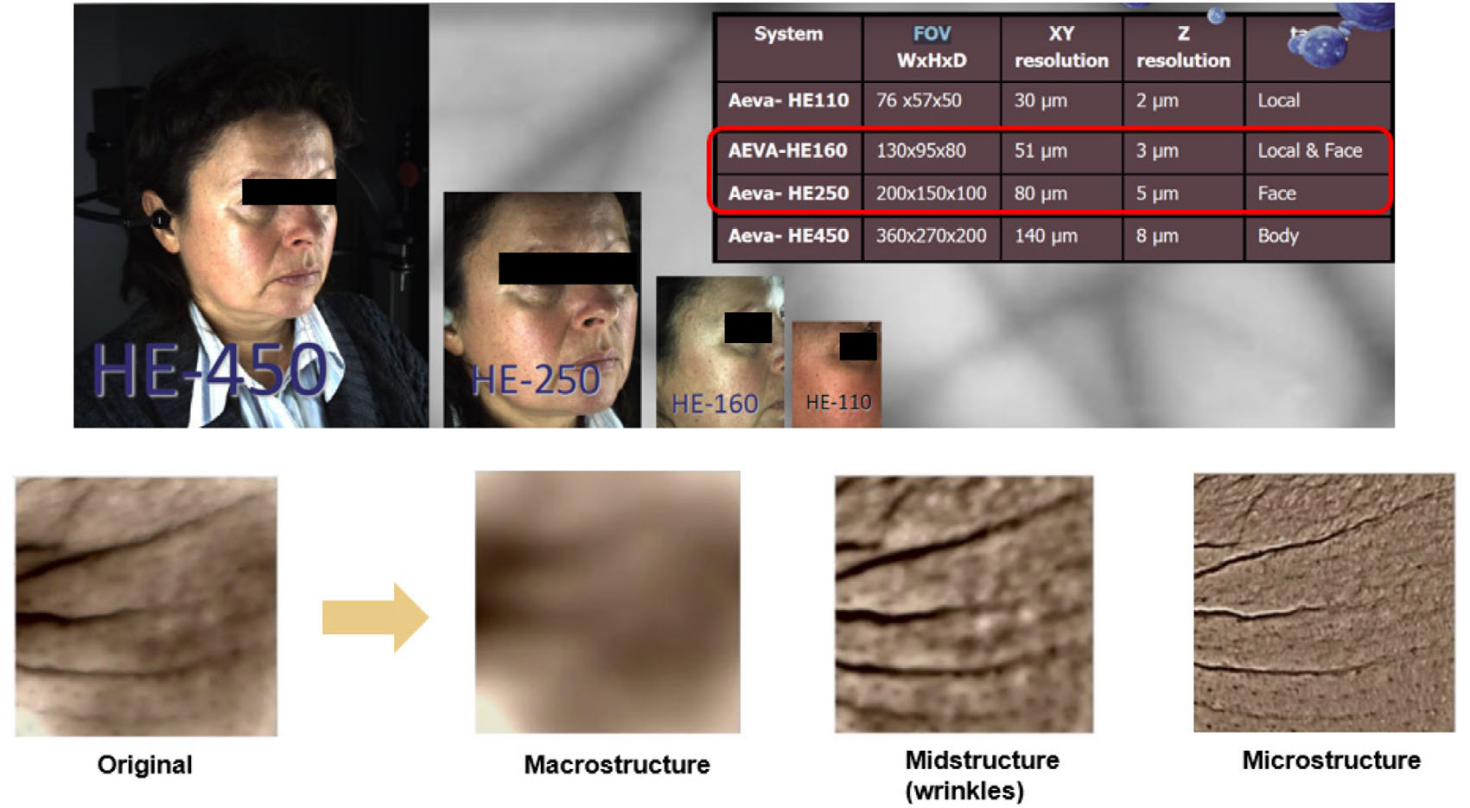
Top: AEVA system field of view (FOV) characteristics. Bottom: Illustrations of microstructure (micro‐relief) and mid structure (wrinkles and fines lines) and their related quantification, obtained in vivo

AEVA‐HE system uses fringes projection combined with stereometry technology and has four possible FOVs (Figure 1). In our study, the focus is made on 160 and 250 FOVs to capture the full face. This technology allows higher resolution cameras and accuracy depends on the pixel distance on the field of view instead of fringes distance. Although techniques based on stereophotogrammetry are of high interest and validated,^23^, ^24^, ^25^ the privilege was given here to fringe projection‐based systems since they provide full field, accurate, and high‐resolution images prone to being further easily processed.

DermaTOP‐HE uses fringes projection technology and captures 3D topographic images with 60 and 120 FOVs.

Both 3D data captured by DermaTOP and AEVA‐HE were visually guided by the AEVA software (AEVA V3, Eotech). All acquisitions were performed in a dark room to avoid altering the contrast between fringes. Image analysis was also performed by the same software AEVA V3, allowing a robust and flexible micro‐structure (micro‐relief) and mid‐structure (wrinkles and fine lines) quantification (Figure 1 given as an example).

### 2.2 In vitro tests

As part of our internal references, a Silflo replica of the crow feet obtained in vivo on a 55 years old woman was taken as an inert model of wrinkles and fine lines that can easily be moved at different angles or positions. As the technical specifications of 3D systems (Field of view, lateral resolution, sensitivity) define their ability to measure zones or skin details of various sizes, two systems were compared, based on the same technology that combines fringes projection and stereometry techniques, that is, the DermaTOP‐HE and the AEVA‐HE. This in vitro approach is indeed crucial in the determination of factors of influence, such as the environmental conditions (the type of measured object), and the repositioning of the system. In addition, in vitro repeatability testing allow defining the capability of the devices in measuring various elements of different sizes with the best accuracy (Gauge, an acceptable compromise between FOV and precision, that is, their limit in the detection of the smallest objects, etc.).

This preliminary phase aims to best evaluate the elements that can be analyzed in further in vivo studies (e.g. post application of an anti‐aging product) with full confidence.

### 2.3 Operational protocol

The standard AEVA software routines and evaluation tools were used as they are commonly applied in clinical tests. These include the DermaTOP ‐HE 3D scanner with FOV 60 and 125, the AEVA‐HE 3D scanner with FOV 160 and 250, the Eotech VisioTOP‐500 positioning bench, and the AEVA V3 software.

A step gauge with calibrated step depths and a skin replica were used for in vitro repeatability testing.

**FIGURE 2:**
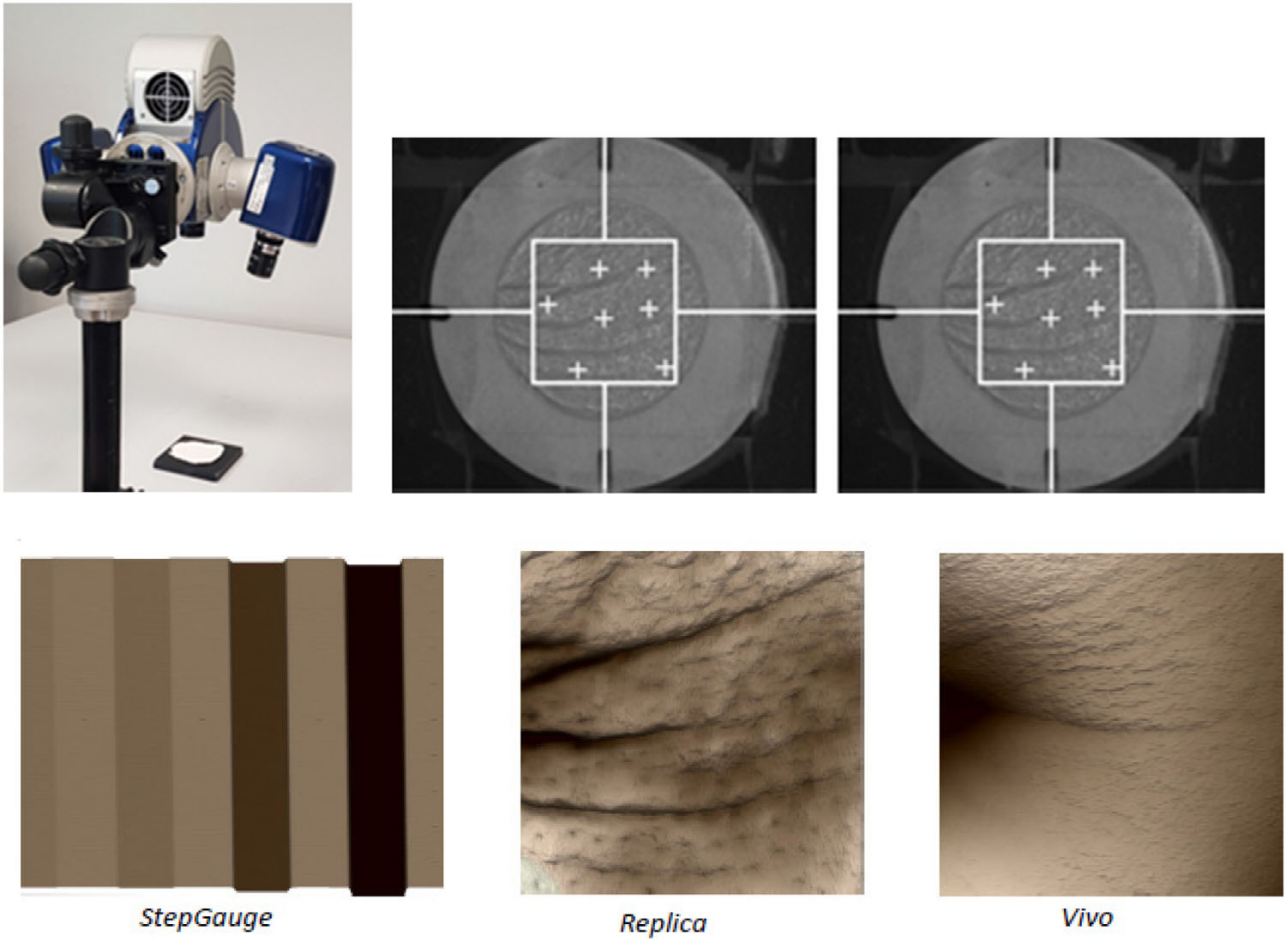
Top: operational conditions used in the positioning and focusing on the skin replica. Down (from left to right): Assessing the step gauge, image of the skin replica, and that obtained in vivo, given as example (see in vivo part)

Using a table stand, the fringes are first oriented perpendicularly to the wrinkles. One operator performed 12 measurements of the Silflo replica and step gauge. These operational conditions are illustrated in Figure 2.

### 2.4 Analytical protocol

3D alignments between measurements were performed between the two systems used AEVA‐HE and DermaTOP‐HE. Both devices used similar settings, that is, an extracted zone of 15 × 15 mm, an LP filter of 0.35 mm, and a Multi‐scale filter of 1.2–3 mm. These technical conditions lead to analysis: i) the Wrinkles Mid‐structure topography, their profile roughness (Rz), and their mean depth, ii) the Microstructure analysis, characterized by its Surface roughness (Stm).

Repeatability deviation is defined as the standard deviation of the 12 measurements on the Silflo replica and step gauge.

## 3 IN VIVO TESTS

### 3.1 Repeatability study (study 1)

This study was conducted in Eotech's offices (suburban Paris) and involved three volunteers with phototypes II to III, seated under comfortable conditions through Visio‐4D benches (photo 1).

Twelve repeated acquisitions for each volunteer were then performed. Volunteers didn't move between measurements.

**Figure:**
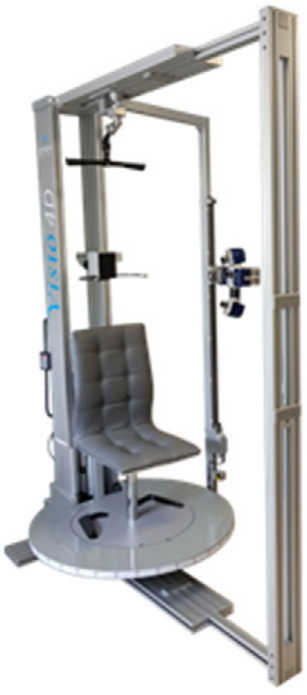
Photo 1 ‐ Illustration of the Visio 4D bench

#### 3.1.1 Collection and analysis of data 

This phase comprised: i) a 3D alignment between measurements, between series and systems, ii) a zone extraction Left and Right, 20 × 20 mm, iii) an LP filter 0.35 mm, and iv) a Multi‐scale filter 1.2–3 mm.

The evaluation of the Mid‐structure topography of wrinkles used the following parameters: the Profile roughness (Rz), and the mean depth of detected objects, the microstructure analysis was assessed by the Surface roughness, Stm.

Repeatability deviation is defined as the mean of the standard deviation of 12 measurements for the 3 volunteers.

### 3.2 Accuracy study (study 2)

#### 3.2.1 Subjects

The study involved 95 Caucasian healthy French women, phototypes II to III, who visited one occurrence of our facilities in suburban Paris. These women were clustered into different age classes 25–39 years (*N* = 7), 40–49 years (*N* = 18), 50–55 years (*N* = 23), 56–60 years (*N* = 25), and 61–71 years (*N* = 22). All women were initially recruited and selected through the visual assessment of crow's feet grade (0–6) by our aestheticians for presenting the largest variability of wrinkles severity. All subjects were informed about the respective purpose and protocol of the study and signed informed consent. The right to imaging was considered non‐applicable since photographs were unseen by all the women under study.  A 1‐day washout was requested for all subjects to avoid the impact of their usual cleansing routines and skin care products prior to the acquisition phase of the images.

#### 3.2.2 Protocol

**FIGURE 3:**
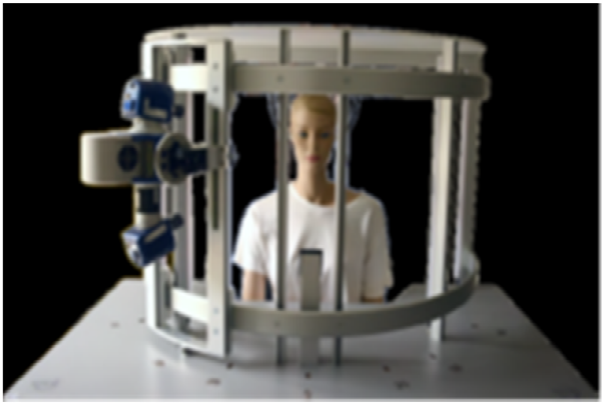
AEVA HE and support table used in the in vivo study 2

**FIGURE 4:**
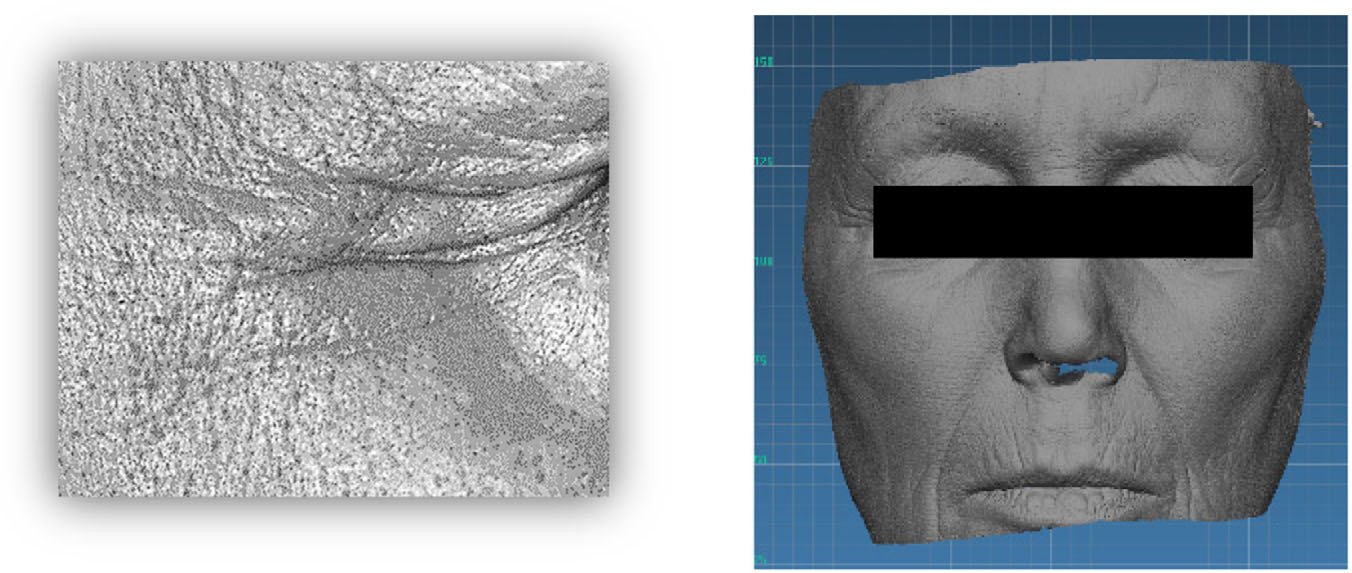
Illustrations of image outputs of the devices. From left to right, DermaTOP‐HE, and AEVA‐HE160

Subjects, once in our facility, rested for 15 min, prior to the imaging phase. The latter comprises the following imaging conditions in both tested systems:

2D and 3D photos of their bare skin were taken with the two devices (Figures 3 and 4):

‐ Full face acquisition using AEVA‐HE (FOV 160)

‐ Left and right crow's feet region acquisition using DermaTOP (FOV 60)

**FIGURE 5:**
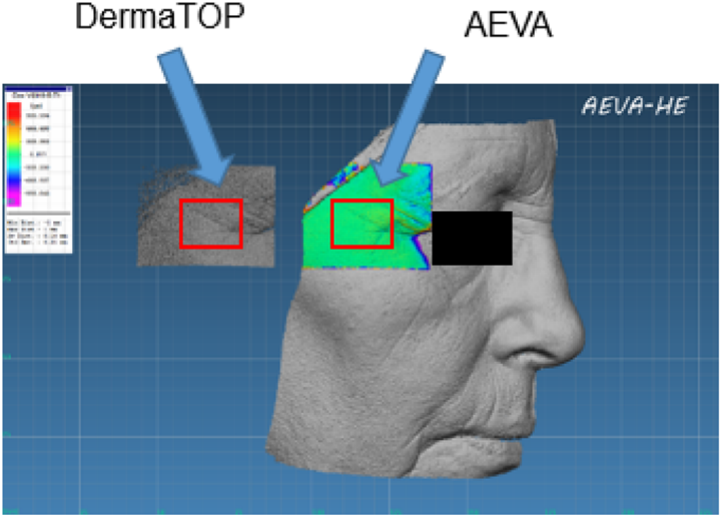
Crow's feet zone extraction from the AEVA‐HE and DermaTOP acquisition on the same woman

AEVA V3 software was used to extract and quantify wrinkles parameters for both AEVA‐HE and DermaTOP. In order to make a fair comparison between both systems, a manual selection of the crow's feet region was performed on the AEVA‐HE data to perfectly fit with that obtained in the region of interest by the DermaTOP (Figure 5). A 20 × 10 mm rectangle area of measurement was positioned.

### 3.3 Statistics

Correlation between the AEVA parameters versus DermaTOP parameters or visual scores at baseline was calculated using the Pearson linear correlation coefficient.

All statistics were performed using IBM SPSS Statistic version 23, and a significance level of α = 0.05 was set throughout the analysis.

The normality of the data was confirmed using the Shapiro‐Wilk normality test. Data were compared using the Tukey test.

## 4 RESULTS

### 4.1 In vitro

#### 4.1.1 Step‐gauge

**FIGURE 6:**
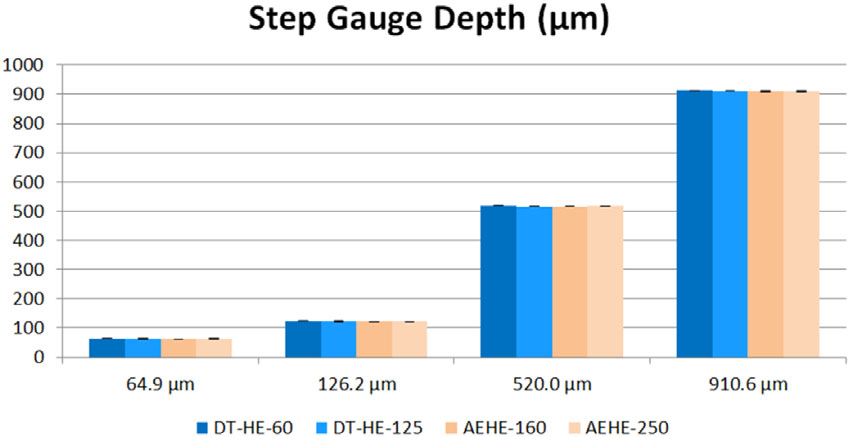
Correlation between DermaTOP‐HE (FOV 60 and 125) and AEVA‐HE (FOV 160 and 250)‐ expressed by the mean depth measurement (μm) on steps

**FIGURE 7:**
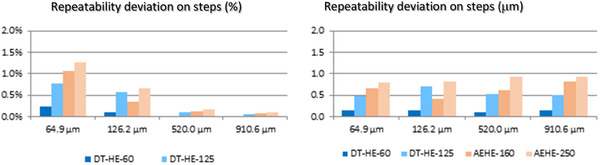
Repeatability deviation on steps for DermaTOP‐HE (FOV 60 and 125,) and AEVA‐HE (FOV 160 and 250)‐ expressed in % at left, and in (μm) at right

Figures 6 and 7 show an excellent agreement between the two devices, with regard to referential gauge values, expressed either as absolute (μm) or %. The relative deviation is <1 μm.

#### 4.1.2 Silflo replica

**FIGURE 8:**
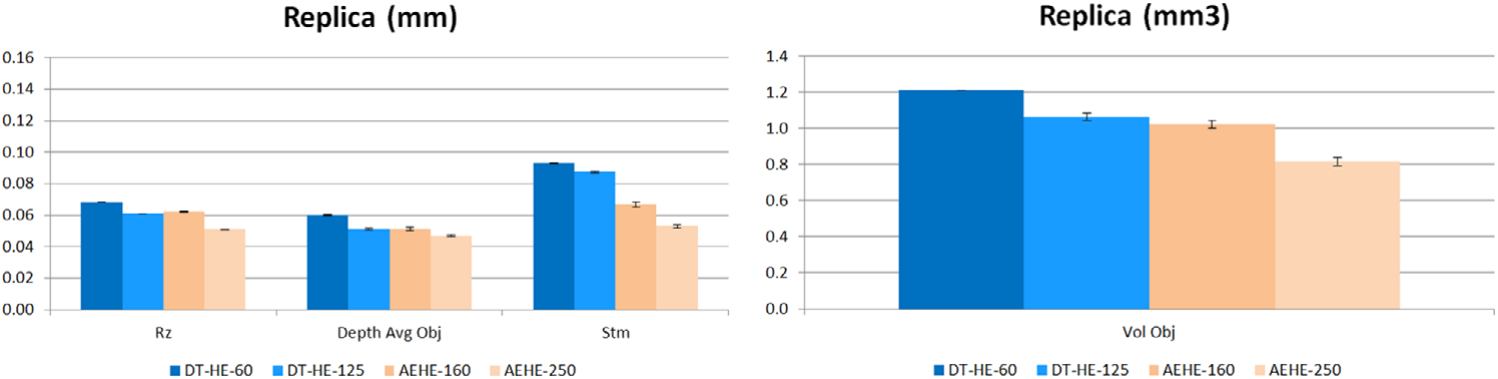
Results on measuring Silflo Replica with DermaTOP‐HE (FOV 60 and 125) and AEVA‐HE (FOV 160 and 250)‐ for Rz, depth and Stm (μm) parameters at left and Volume (mm3) parameter at right

**FIGURE 9:**
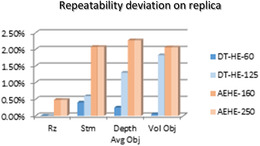
Repeatability deviation on Replica for DermaTOP‐HE (FOV 60 and 125,) and AEVA‐HE (FOV 160 and 250) expressed in %

As shown by Figures 8 and 9, an excellent agreement was found between both devices, either in measuring size or the volume of the wrinkles, and repeatability reached a highly acceptable level (about 97.5%). In short, all quantified values are in highly fair agreement between the two systems.

### 4.2 In vivo

#### 4.2.1 Repeatability of AEVA‐HE versus DermaTOP‐HE in study 1

**FIGURE 10:**
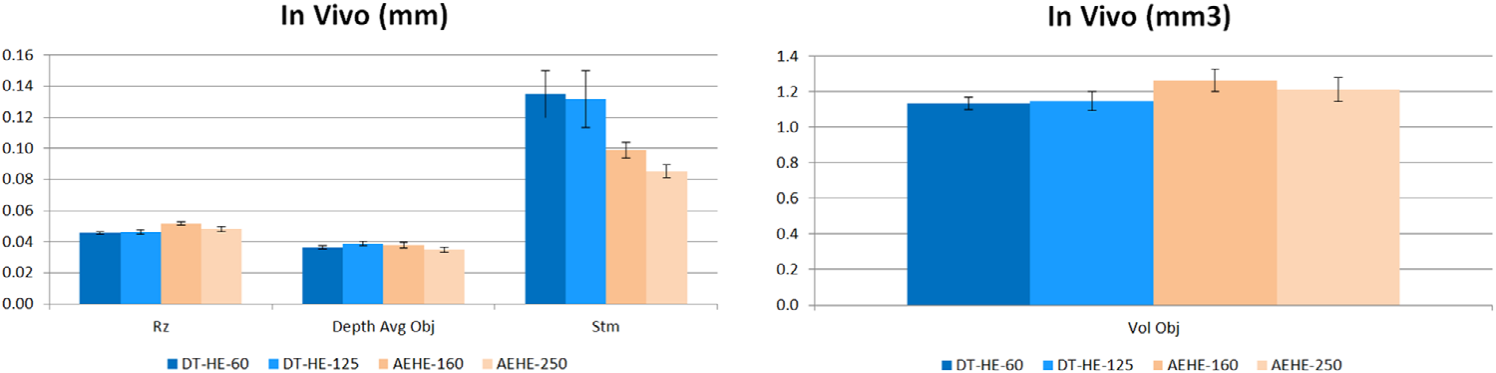
Results on measuring in vivo with DermaTOP‐HE (FOV 60 and 125) and AEVA‐HE (FOV 160 and 250)‐ for Rz, depth and Stm (μm) parameters (left) and Volume (in mm^3^) parameter (right)

**FIGURE 11:**
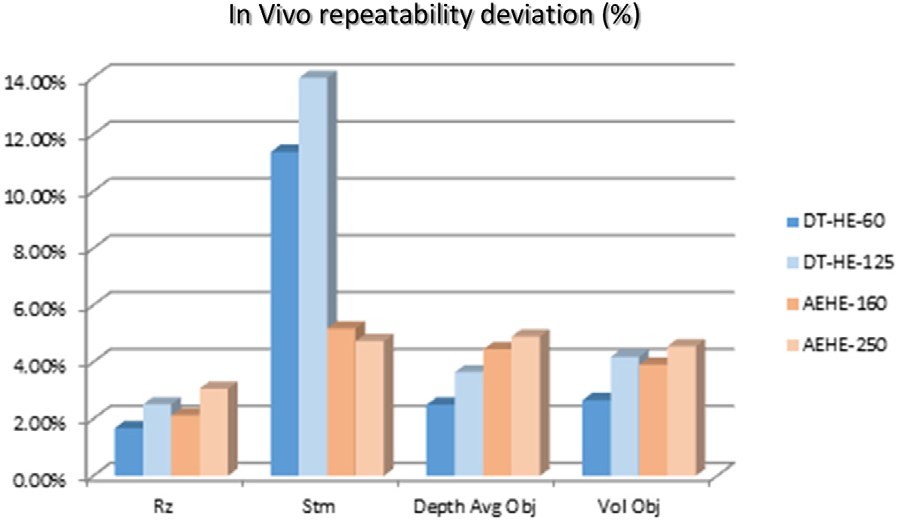
Repeatability deviation on vivo for DermaTOP‐HE (FOV 60 and 125,) and AEVA‐HE (FOV 160 and 250) expressed in %

As shown by Figures 10 and 11, both devices, using two different filters, present a high repeatability level, in vivo, in the quantification of wrinkles size (in mm) or volume (mm^3^) of 95% or above. However, the Stm value appears of a much higher value in the case of the DermaTOP‐HE system, about twice that obtained by the AEVA‐HE device.

#### 4.2.2 Performances of AEVA‐HE versus DermaTOP in study 2

**FIGURE 12:**
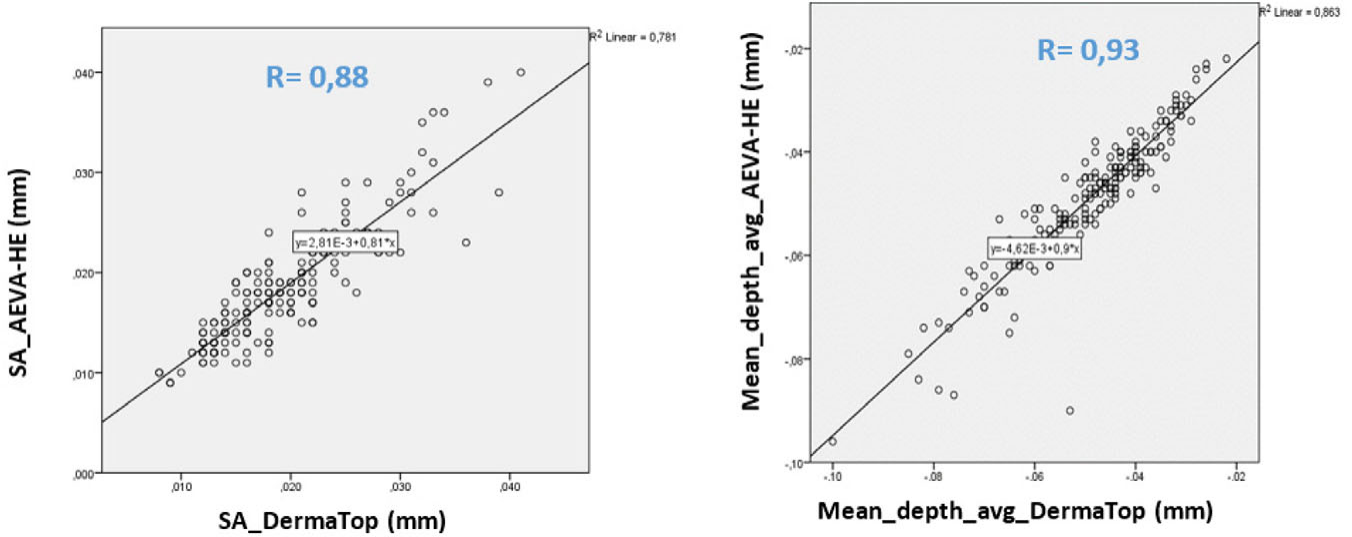
12.a (left) and 12.b (right) showing the highly significant correlations between both systems in the quantification of SA, expressed in mm (Figure 12) and that of the mean depth of wrinkles (Figure 13)

**FIGURE 13:**
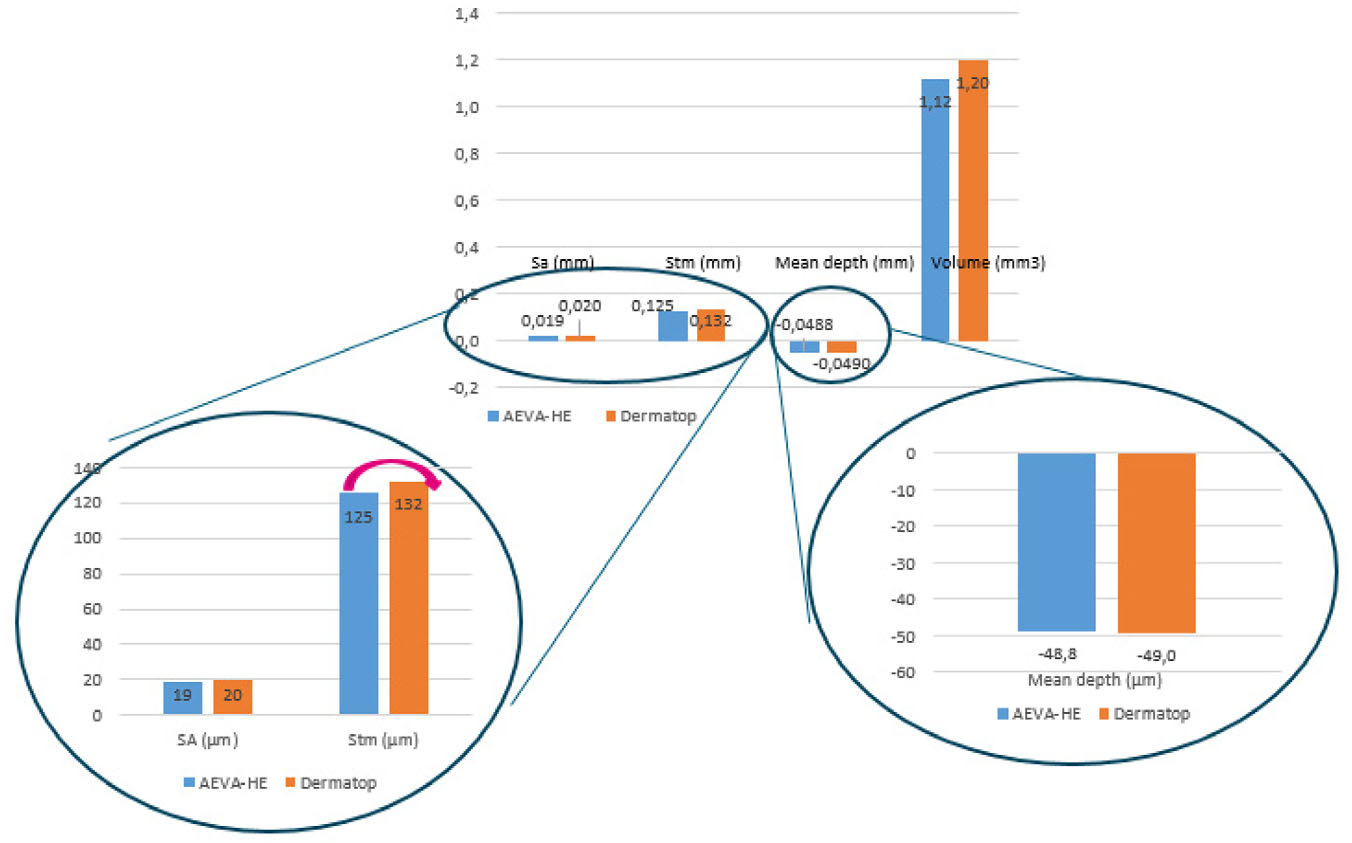
Statistical results of the comparison between AEVA and DermaTOP

When focusing on the crow's feet region extracted from both systems, using the same region of interest dimensions, comparable and highly significant results are obtained (Figures 12, 13).

“Sa” roughness parameter, (*r* = 0.88) and for mean depth parameter (*R* = 0.93) with *p* < 0.000001 in both cases. Such is not the case with Stm, suggesting that the DermaTOP‐HE is more sensitive to microstructure than AEVA due to the blue light and a higher optical resolution.

## 5 DISCUSSION/CONCLUSION

Measurements of facial signs, especially for anti‐aging effects upon wrinkles and facial sagging are not only crucial to cosmetic researchers but own precious and ethical respect to consumers, by best illustrating and/or sustaining the anti‐aging claim. The age‐related changes in temporary and persistent facial skin wrinkling occur very slowly over a person's lifetime.

The AEVA software can either measure, locally or globally, a face and extract any zone of interest (crow's feet, eye bags, forehead, peri‐oral, glabella or nasal folds, and neck). Local zone analysis of skin microstructure includes pores, fine lines & wrinkles evaluation. Objective parameters can be calculated from each of these zones such as the number, depth, or volume of wrinkles and folds. In this validation study, the repeatability of this new system was investigated and the accuracy

of the local measurements it generated was determined. The present article focused mainly on persistent wrinkles in a static face. Temporary wrinkles resulting from a facial expression could be the object of extensive work.

The present work showed that the new AEVA‐HE device, of high robustness, and easy and fast use, affords very similar data to those provided by the DermaTOP‐HE. Not only did in vitro tests assessed

the high repeatability and accuracy of the measurements given by the AEVA system, but in vivo tests showed a high correlation between AEVA‐HE and DermaTOP‐HE devices, with the exception of surface roughness (Stm).

As a logical second step, it has been utilized in the follow‐up of the effects brought by anti‐aging products still in development, that is, ensuring a screening phase. The results of such an applied study will be reported in a subsequent paper (Ibid).

The developed method can objectively and accurately evaluate some physical parameters of wrinkles and their facial density. As an investigated model in the present work, Its practical organization which needs a fully equipped room is likely a limiting factor in the daily follow‐ups of large cohorts of subjects. In such cases, portable devices, now available, are probably to be preferred.

One of the interesting next steps would be to segment wrinkles and track, in time, each wrinkle object by measuring their length, width, depth, and spatial distribution following the work of Decenciere et al.^26^ Global face topology analysis with the Aeva software, comprising re‐pulping, firming, oval and sagging, could be addressed in a future work dealing with holistic face evaluation and its link with perceived clinical scores.

## FUNDING INFORMATION

All costs of the study were met by the L'Oréal Research and Innovation Department.

## CONFLICT OF INTEREST

The authors are either employees of the L'Oréal and Research Innovation Department or the Eotech Company.

## Data Availability

Research data are not shared.
